# Pricing of Staple Foods at Supermarkets versus Small Food Stores

**DOI:** 10.3390/ijerph14080915

**Published:** 2017-08-15

**Authors:** Caitlin E. Caspi, Jennifer E. Pelletier, Lisa J. Harnack, Darin J. Erickson, Kathleen Lenk, Melissa N. Laska

**Affiliations:** 1 Department of Family Medicine and Community Health, University of Minnesota, Minneapolis, MN 55455, USA; 2 Office of Statewide Health Improvement Initiatives, Minnesota Department of Health, St Paul, MN 55164, USA; Jennifer.Pelletier@state.mn.us; 3 Division of Epidemiology and Community Health, University of Minnesota, Minneapolis, MN 55455, USA; harna001@umn.edu (L.J.H.); erick232@umn.edu (D.J.E.); lenk@umn.edu (K.L.); nels5024@umn.edu (M.N.L.)

**Keywords:** corner stores, food affordability, food deserts, health disparities

## Abstract

Prices affect food purchase decisions, particularly in lower-income communities, where access to a range of food retailers (including supermarkets) is limited. The aim of this study was to examine differences in staple food pricing between small urban food stores and the closest supermarkets, as well as whether pricing differentials varied based on proximity between small stores and larger retailers. In 2014, prices were measured for 15 staple foods during store visits in 140 smaller stores (corner stores, gas-marts, dollar stores, and pharmacies) in Minneapolis/St. Paul, MN and their closest supermarket. Mixed models controlling for store type were used to estimate the average price differential between: (a) smaller stores and supermarkets; (b) isolated smaller stores (>1 mile to closest supermarket) and non-isolated smaller stores; and (c) isolated smaller stores inside versus outside USDA-identified food deserts. On average, all items except white bread were 10–54% more expensive in smaller stores than in supermarkets (*p* < 0.001). Prices were generally not significantly different in isolated stores compared with non-isolated stores for most items. Among isolated stores, there were no price differences inside versus outside food deserts. We conclude that smaller food stores have higher prices for most staple foods compared to their closest supermarket, regardless of proximity. More research is needed to examine staple food prices in different retail spaces.

## 1. Introduction

For over a decade, researchers have noted persistent disparities in access to healthy food in urban areas of the U.S. [1,2,3,4,5,6,7,8,9]. In census tracts with high proportions of minority or low-income residents, supermarkets are scarce and convenience stores and fast-food outlets are abundant [7]. Beyond the presence of different types of stores, low-income neighborhoods have demonstrated lower fruit and vegetable availability [10,11] and lower overall healthy food availability [12]. A growing concern about the impact of ‘food deserts’ on dietary quality and obesity has sparked nationwide efforts to identify priority areas for food environment interventions. According to the U.S. Department of Agriculture (USDA), food deserts are defined as census tracts that are low-income (a specified proportion of residents fall below an income threshold) and low-access (a specified proportion of residents have poor access to a supermarket) [13]. Residents of food deserts face constrained food choices by the concurrence of both economic and physical obstacles [14]. Residing in a USDA-defined food desert has been associated with indicators of poor health, including higher rates of obesity [15], lower levels of serum carotenoids, and higher blood pressure [16].

While the presence of food deserts across the U.S. has been well established, few studies have examined potential differences in food prices in various food retail spaces [17]. This is important to understand, as pricing differences influence food purchasing decisions [17,18,19]. In general, supermarkets obtain food at wholesale prices and have more efficient economies of scale than smaller food stores, resulting in lower prices [9,20,21]. In a recent national study, Rimkus et al. examined milk prices across a number of store types and found prices were significantly lower at supermarkets than at limited service stores [22]. Nevertheless, price variability for the same product does not always follow expected patterns [23,24], as several studies have demonstrated lower prices for fresh foods at small food stores than at supermarkets [25,26,27]. If food deserts lack supermarkets, residents of food deserts might face higher prices simply because of the types of stores available in low-income neighborhood.

Looking beyond store type, a store’s geographic location is relevant to pricing. Stores that are geographically isolated from other food stores may carry more expensive inventory. Although recent U.S. studies have generally not found food to be more expensive in low-income neighborhoods [22,23,28,29], food stores located far from a competitor may set prices higher than food stores located near supermarkets, which may need to offer more competitive pricing [9,20]. This relationship has not been well studied in the U.S. context. In an Australian study, having a greater number of major supermarket chains within 5 km of small stores was associated with a lower cost of healthy food [30]. These patterns could have particular implications for residents of food desert. Already economically and geographically constrained, they may also face higher prices due to store isolation.

The purpose of this study was to assess differences in staple food item prices in small and non-traditional food stores (hereafter “smaller food stores,” including corner stores, gas-marts, pharmacies, and dollar stores) compared to supermarket prices, and how pricing patterns could exacerbate the problem of food deserts. Our aims were to: (1) compare prices of staple foods at smaller food stores with prices at their closest supermarkets; (2) compare prices of staple foods in smaller food stores more isolated (>1 mile) versus less isolated (≤1 mile) from supermarkets; and (3) examine prices of isolated smaller food stores inside and outside of food deserts. We hypothesized that prices would be higher in smaller food stores compared to supermarkets, while prices in isolated stores would be higher than in non-isolated stores. We had no reason to expect that higher prices would be evident in isolated food deserts versus non-food deserts, but because even moderate price differences may have significant consequences for low-income residents, we examined store isolation pricing patterns specifically in food deserts.

## 2. Materials and Methods

### 2.1. Study Design and Sample

#### Study Overview

We collected data in the fall of 2014 as part of the baseline evaluation of a city ordinance regulating minimum stocking requirements for food retailers in Minneapolis, MN, as well as comparable stores in St. Paul, MN (the control site). To improve the availability of healthy items across all neighborhoods, the Minneapolis Staple Foods Ordinance applied to all licensed grocery stores [31], with specific exceptions outlined in the Supplementary Materials. The ordinance [32] was implemented in April 2015 and outlines specific stocking requirements for 10 different staple food categories (e.g., fruits and vegetables, whole grains). The focus of the evaluation was on stores that would likely struggle to meet the ordinance requirements.

We obtained lists of stores from the Minneapolis Health Department for Minneapolis stores and the Minnesota Department of Agriculture for St. Paul stores. We refined the licensing agency lists to include only Minneapolis stores eligible for ordinance enforcement as well as comparable stores in St. Paul. Stores exempt from the ordinance were excluded from the study. Reasons for exemption are detailed in the Supplementary Data. They included stores that were “accessory use” because they: (1) sold staple foods only as an accessory to its primary business, or (2) sold only specialized classes of foods and would not be expected to stock food in many staple food categories (e.g., produce stands, spice shops). The accessory use exemption did not include SNAP-authorized stores, as these stores would be expected to stock food in several staple food categories per SNAP requirements. Most gas-marts, dollar stores, and pharmacies accepted SNAP and were thus eligible for ordinance enforcement and eligible for inclusion in this study. The ordinance also excluded stores located in the core downtown commercial district. For the evaluation study, we also excluded stores expected to exceed minimum stocking requirements, including supermarkets and stores listed in the state database as participating in the Special Supplemental Program for Women Infants and Children (WIC). Out of 255 eligible smaller stores, we randomly selected 180 to participate. After completing in-person visits, 23 were ineligible (e.g., due to new participation in WIC) and 17 others did not give active consent for a store assessment. The remaining 140 stores could be classified as corner or small grocery stores (*n* = 58), gas-marts (*n* = 47), dollar stores (*n* = 13), pharmacies (*n* = 21), and one general retailer.

To identify closest supermarkets, we used a multi-stage approach to identify a comprehensive list of supermarkets located nearby smaller food stores. This method was meant to include all stores that functioned as supermarkets in the community even if they were not chain stores. Using area-specific knowledge and “boots on the ground” assessments is a recommended practice in the field [33,34,35], superior to relying only on business listings, which have been shown to be prone to error and systematic missingness [36,37,38]. For example, relying exclusively on administrative business data could result in the systematic exclusion of stores that are perceived by the local community to be supermarkets, but are not identified as such on lists. To avoid these problems, our process included four steps. First, we supplemented city-level administrative stores lists with ESRI Business Analyst data for all stores located within 2 miles of any smaller store (including those outside Minneapolis and St. Paul boundaries) in order to avoid omitting supermarkets that were just outside city limits [33]. Second, from these lists, the study team generated an initial list of possible supermarkets that included both chain supermarkets as well as food cooperatives, mass merchandisers (e.g., Super Target), and wholesaler clubs. The team sought to include stores that met all three of the following criteria: (1) had grocery carts; and (2) had at least three cash registers; and (3) were qualitatively similar to a supermarket based on its overall environment, range of products, and customer use for purchasing groceries. Third, for face validity, six local food environment experts, familiar with the study area through their work as academic food environment researchers, local public health professionals and those with food retail industry experience, reviewed the list of stores in the study area and suggested additional stores that might meet the three criteria for a supermarket. Fourth, trained study staff conducted site visits to determine whether each store met the criteria.

We identified 76 supermarkets in the study area. Using ArcGIS, we calculated the distance between each small store and the closest supermarket. We also identified the second closest supermarket. We matched smaller food stores to the closest supermarket that sold each food item, which included 44 unique supermarkets.

### 2.2. Measures

Data collectors recorded price data for 54 food and beverage items at smaller food stores and supermarkets. The data collection instrument was based on a form developed by the Yale Rudd Center to evaluate changes in WIC packages [39] and included milk, fresh and frozen fruits and vegetables, cereal, bread, rice, tortillas, eggs, canned tuna, peanut butter, and dry beans. Data collectors recorded the price of fruits and vegetables (per item or per pound), which was converted to price per pound in the analysis. They recorded milk prices for half-gallon and gallon size containers, and eggs prices for one-dozen containers. For other items, data collectors looked for designated packages sizes and recorded the lowest unit price for that size, excluding sale prices. They also recorded if a food was not present or if a price could not be obtained. If items were not available in the designated package size, they recorded the lowest unit price in the closest size. Then, in the analysis, we converted small food store items to the following common package sizes if they were not already in these sizes: for cereal (per 18 oz), bread (per 20 oz), rice and dry beans (per 16 oz), canned tuna (per 5 oz), and peanut butter (per 17 oz). Size standardization was commonly necessary for grain-based items and dry beans (26% to 82% of small stores) as these items have many common package sizes, but standardization was rarely necessary (<10%) for tuna and peanut butter.

We calculated the street-network distance between smaller stores and their closest supermarket. Smaller stores were defined as isolated if the distance was >1 mile to the closest supermarket. We selected 1 mile because 0.95 miles was the median distance between smaller stores and supermarkets in our sample. Moreover, previous research in urban areas has shown that residents travel approximately 1 mile for small shopping trips [40], and low-income residents travel 1–1.5 miles for their main shopping trip [41].

We used USDA criteria [13] to determine whether smaller stores were located in low-income and low-access census tracts. Tracts were classified as low-income if they met any of the following criteria: (1) median family income ≤80% of the state-wide median family income; (2) poverty rate >20%; or (3) median family income s ≤80% of the metropolitan area’s median family income. Census tracts met the criteria for low-access if ≥100 households were >1/2 mile from the nearest supermarket and had no access to a vehicle. While the food desert status variable includes a dimension of isolation, the two variables are conceptually different and preliminary analyses indicated that there was limited overlap between them (*r* = 0.21). We identified census tracts meeting these criteria using the USDA Food Access Research Atlas [13]. We then classified smaller stores according to their location inside or outside food deserts.

### 2.3. Analysis

In descriptive analyses, we calculated the availability and average price of items at smaller stores and supermarkets. Chi-square tests (3 df) tested whether availability of items differed across types of smaller stores. Price differentials, representing the difference in price between each item at smaller stores and supermarkets, were calculated in two ways: (1) the difference between overall average smaller store price and overall average supermarket price; and (2) the average difference between each smaller store and its corresponding closest supermarket. We used the former for descriptive purposes, whereas we used the latter to model relationships between store characteristics and prices. Price differentials were expressed as a percent difference of the supermarket price.

In mixed models, in which the unit of analysis was the smaller store-supermarket pair, we tested the association between store isolation and price differential for each item. In the fixed portion of the model, we included a dichotomous measure of store isolation, a dichotomous measure of food desert status, an interaction term between the two, and dummy variables to control for smaller store type, excluding the general retailer. The random portion of the model included a census tract indicator to account for stores clustering within census tracts. We used the results of the models to calculate predicted values for price differentials between smaller stores and supermarkets in isolated and non-isolated census tracts (averaged over food desert status) and in food desert and non-food desert census tracts (holding isolation status constant at “isolated”) using marginal effects. Results estimate the average price differential across the four smaller store types as a percent difference from supermarkets. Data were analyzed in 2016 using Stata version 13.1 (College Station, TX, USA).

## 3. Results

The analysis was limited to items carried by at least 40% of smaller stores, so that item-specific comparisons could be made between smaller stores and their closest supermarkets. Consequently, some healthy items for which we obtained data (e.g., whole wheat bread, brown rice) were excluded from the analysis. The final list of 15 items included three varieties of fruits and vegetables, four varieties of milk, four varieties of protein staples, and four varieties of grain staples. All four varieties of milk (skim, 1%, 2% and whole) were common in both gallon (3.78 L) and 1/2-gallon (1.89 L) containers; since results were similar across sizes, we present only gallon prices for the milk varieties.

Availability of food and beverage items varied by store type (Table 1). There was considerable variability of items across smaller store type. Across all smaller stores, the most common items were peanut butter (80%), eggs (79%), white bread (78%), tuna (76%), and 2% milk (75%).

### 3.1. Aim 1: Comparing Prices at Supermarkets versus Smaller Stores

Table 2 presents the average price differential between smaller stores and supermarkets for each item. Price differentials are presented relative to the supermarket price. For example, results show that the price of milk was 11–14% higher in small food stores relative to supermarkets, depending on the milk variety.

On average, all items were more expensive in smaller stores than in supermarkets except white bread, which was more expensive in supermarkets. The items with the highest price differential were Cheerios^TM^ cereal (54%), bananas (53%), and white rice (50%).

### 3.2. Aim 2: Comparing Prices at Smaller Stores Isolated from Supermarkets versus Those That Are Not Isolated

**Figure 1:**
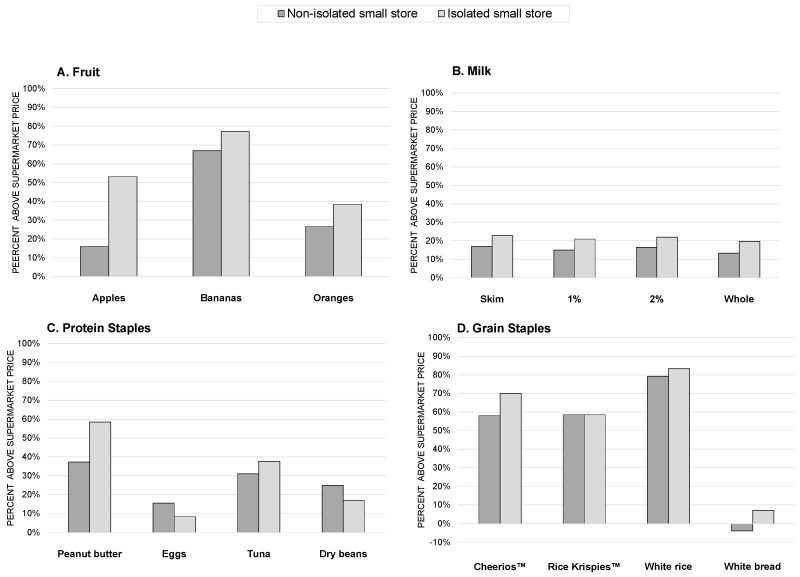
Price Differentials for Items at Small Food Stores by Store Isolation Status, Minneapolis and St. Paul, MN, 2014.

Figure 1 presents the price differentials between smaller stores and supermarkets, according to smaller store isolation status. For all items except eggs, dry beans, and Rice Krispies^TM^, the direction of these comparisons was the same, reflecting greater price differences in isolated stores than non-isolated stores. However, apples were the only item for which the price differential was statistically significantly greater in isolated stores compared with non-isolated stores (*p* = 0.01).

### 3.3. Aim 3: Examining Prices of Isolated Smaller Stores Inside and Outside of Food Deserts

**Figure 2:**
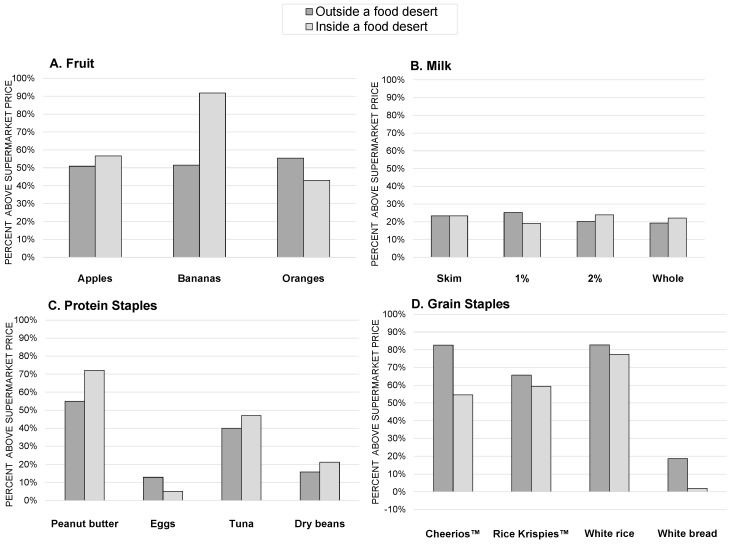
Price Differentials at Isolated Small Food Stores by Food Desert Status, Minneapolis and St. Paul, MN, 2014.

Figure 2 presents the price differential for isolated smaller stores located inside and outside of food deserts. There was no statistically significant difference in price for any item. Of the 15 items, price differentials were higher inside food deserts for 7 items, the same for 1 item, and lower for 7 items.

## 4. Discussion

Most of the staple food items examined in this study (14 of 15) were priced higher at smaller stores than supermarkets (range 6% to 54% higher in price). Smaller stores have limited distribution of perishable items and smaller economies of scale than supermarkets, which manifests itself in higher food supply costs [20]. For instance, Minneapolis and St. Paul smaller stores mostly acquire produce through independent purchases from a larger store/wholesaler versus a distributor [42], which may increase costs to consumers. Smaller stores also tend to sell items in smaller package sizes than supermarkets, so these items generally have a greater price per unit. Historically, chain supermarkets have been more likely to carry generic items than other store types [20], although this is possibly an area where the food retail landscape is changing, as non-traditional food retailers like pharmacies have begun offering their own brands of foods. The observable price differences between smaller stores and supermarkets may contribute to customer purchasing decisions. Research has indicated that price elasticity for specific food products can be high [43,44,45,46], assuming stability of other product categories. By one estimate, a 10% decrease in the price of fruit was estimated to increase purchasing by 7% [44]. However, it would be prudent to interpret this estimate as an upper bound, as the elasticity for any particular price point does not reflect the entire demand curve.

Descriptive findings in this study showed that isolated smaller stores had higher prices than non-isolated stores for 12 out of 15 staple food items. However, only one item (apples) was statistically significantly more expensive. Given our small sample size, which limits statistical power, more research is needed to understand pricing of healthy and staple foods in isolated areas. Another area for future research is whether price differentials are more pronounced for more healthful compared to less healthful foods. If prices of less healthful food are also elevated in smaller stores, higher prices for staple items might have no net effect on the nutritional quality of foods purchased due to reduced purchasing power for all food types.

The prices of items at smaller isolated food stores were similar inside and outside of food deserts. While no objective pricing disparity was observed by food desert status, the public health relevance of price differentials is particularly salient in food deserts. By definition, in food deserts, a high proportion of residents are low-income, making them more price sensitive [17]. As discussed in several qualitative and mixed-methods studies, decisions around food shopping may be particularly complex in low-income households, incorporating economic and geographic realities as well as other personal, cultural, or demographic considerations [14,47]. Households with young children may be particularly attuned to prices when making shopping decisions [14]. In a qualitative study of low and mixed-income residents in Philadelphia, price was the top consideration in deciding where to purchase staple food; distance to the store was noted as an important constraint only among residents who did not have a car [19]. Residents without vehicle access are either limited to small local food stores for grocery shopping or rely on alternate transportation, which adds both travel costs and time [48]. In one study of food access in Bridgeport, CT, Leclair and Aksan contend the true cost of traveling from a food desert to a supermarket (including both travel and opportunity costs) is $5–7 [49]. Research in the Minneapolis and St. Paul, MN metropolitan area has shown that low-income residents frequently pay for transportation to more remote food establishments, including taxicabs and informal taxis, because of limited personal vehicle access [48]. While proximity is only one factor influencing shopping decisions [50,51], geographic constraints in low-income and low-access areas are not as relevant for higher-income households with vehicle access.

Reliance on smaller food stores for household food supplies may also contribute to food insecurity in food deserts. Higher prices in smaller stores mean people are able to buy less food with their limited resources. Many smaller food stores accept Supplemental Nutrition Assistance Program (SNAP) benefits (95% in our smaller food store sample), and unlike WIC-authorized stores, in which prices for WIC-allowed foods are capped at 115% of the state average costs [52], SNAP-authorized stores have no such limits. Thus, when SNAP benefits are used on staple foods in smaller food stores, they may not stretch as far. To compensate for reduced purchasing power in smaller food stores, policymakers could consider capping prices for designated staple foods in SNAP-authorized retailers, or increasing SNAP incentives for families residing in food deserts.

Results are also relevant in determining the success of the Minneapolis Staple Foods Ordinance, which requires small food stores to stock minimum amounts and varieties of staple foods, but makes no reference to prices. The study presents a unique opportunity to examine possible price differences in staple food as they change over time. As availability of healthy food expands to a new retail spaces, policy success will require that the expanded inventory in small stores is affordable. While availability is likely to increase, it remains unseen how prices will be affected, particularly in low-income/low-access areas of the city.

In interpreting results, one consideration is that when pre-specified sizes were not available, data collectors gathered data on the closest package size instead, so sizes needed to be standardized for comparison. This was common for grain items and dried beans. For example, in smaller stores the average package size for Cheerios^TM^ cereal was 22% smaller than in supermarkets. Comparison of non-like package sizes is a less conservative approach than if we had only compared precisely equivalent sizes. Non-like comparisons reflect a difference in product availability across two stores; thus, we cannot assume that, if the stores hypothetically stocked the exact same brand or size item, that the prices would be different. Nevertheless, as noted by Kaufman et al. (1997), our method is a better reflection of the lowest price available to the consumer than an approach that compares only precisely equivalent items [20]. When stores stock similar (but not identical) items, the difference in availability is manifested in a difference in price, which is relevant to the consumer and the likelihood of purchase. Furthermore, differences in package sizes were not always as expected. For example, dry beans and white rice had average package sizes that were larger in the smaller stores than supermarkets.

The study has several other limitations. The focus of this research was on the price differentials in different store types, but the effect of those differentials on food purchase decisions must be explored in future work. The study also did not include WIC stores, which may be source of competition for smaller stores in this study sample. However, inclusion of WIC stores in pricing studies presents unique challenges, as WIC products are capped at 115% of the state average cost, and vendors may set prices differently when customer are likely to pay with WIC vouchers. Finally, USDA food deserts do not align perfectly with other measures of healthy food access. Food desert status can be defined by many criteria, which may yield different boundaries [53,54]. Thus, results could vary if alternate criteria were used to define low-income/low-access areas.

## 5. Conclusions

Taken in context with other studies, our results support the notion that, for staple food items commonly stocked in smaller food stores, prices are generally higher in smaller stores compared with supermarkets. More research utilizing a health equity perspective is needed to examine grocery prices in different retail spaces, especially in food deserts where high food prices could contribute to food insecurity. This research may be particularly relevant to the discourse on the upcoming Farm Bill reauthorization in 2018, which addresses SNAP retailer authorization requirements. Such work can also assist in identifying unanticipated consequences of the Minneapolis Staple Foods Ordinance or other local policies related to minimum stocking of healthy food. Future research should also examine the effect of pricing differentials on objective measures of shopping behaviors in quasi-experimental studies.

## Figures and Tables

**Table 1:** Availability of items ^a^ at supermarkets, small food/corner stores, gas-marts, dollar stores, and pharmacies, Minneapolis and St. Paul, MN, 2014.

Item	Supermarkets (*n* = 44) ^b^	All Smaller Food Stores (*n* = 140)	Small Grocery/Corner Store (*n* = 58)	Gas-Mart (*n* = 47)	Dollar Store (*n* = 13)	Pharmacy (*n* = 21)	*p*-Value
	%	%	%	%	%	%	
*Produce*							
Apples	100	49	47	80	0	14	<0.001
Bananas	100	58	58	94	0	14	<0.001
Oranges	98	42	43	62	0	18	<0.001
*Milk*							
Skim	91	63	35	86	38	95	<0.001
1%	82	62	35	92	31	82	<0.001
2%	95	75	57	98	38	86	<0.001
Whole	95	74	61	94	38	77	<0.001
*Protein staples*							
Peanut butter	95	80	65	88	85	95	0.004
Eggs	86	79	78	86	62	73	0.229
Tuna	93	76	59	86	85	91	0.002
Dry beans	98	42	76	12	92	0	<0.001
*Grain staples*							
Cheerios^TM^	72	56	43	58	54	86	0.006
Rice Krispies^TM^	64	41	37	26	38	82	<0.001
White rice	100	59	85	30	46	67	<0.001
White bread	93	78	70	86	62	86	0.087

^a^ Items in column include those present in >40% of stores. *p*-value is 3 df test across smaller store types; ^b^ Unique supermarkets that were identified as the closest to each smaller store.

**Table 2:** Price differences between small food stores and nearest supermarkets with item available for 15 products in Minneapolis and St. Paul, MN, 2014.

Item	N (Store Pairs)	Average Price at Small Food Store	Lowest, Highest Small Food Store Price	Average Price at Nearest Supermarket	Lowest, Highest Supermarket Price	Price Differential Relative to Supermarket Price (%)	*p*-Value
*Produce*							
Apples (lbs)	56	$1.94	$1.09, 4.20	$1.76	$0.89, 2.99	10%	<0.001
Bananas (lbs)	64	$1.18	$0.49, 1.93	$0.77	$0.29, 1.39	53%	<0.001
Oranges (lbs)	42	$2.03	$0.79, 3.55	$1.75	$0.50, 2.99	16%	<0.001
*Milk*							
Skim (gallon)	77	$4.05	$3.49, 5.79	$3.54	$2.42, 4.99	14%	<0.001
1% (gallon)	76	$4.09	$2.19, 3.39	$3.60	$1.72, 2.99	14%	<0.001
2% (gallon)	93	$4.18	$2.19, 3.99	$3.70	$1.79, 3.39	13%	<0.001
Whole (gallon)	90	$4.28	$2.19, 3.99	$3.86	$1.79, 2.99	11%	<0.001
*Protein staples*							
Peanut butter (17 oz)	112	$3.89	$1.00, 5.99	$3.00	$1.21, 5.33	30%	<0.001
Eggs (dozen)	87	$2.43	$1.00, 4.29	$2.27	$1.39, 3.49	7%	<0.001
Tuna (5 oz)	105	$1.68	$0.80, 2.49	$1.58	$0.65, 3.99	6%	<0.001
Dry beans (16 oz)	57	$1.86	$0.99, 2.99	$1.64	$0.60, 2.59	13%	<0.001
*Grain staples*							
Cheerios^TM^ (18 oz)	70	$7.41	$2.16, 11.10	$4.82	$3.54, 7.94	54%	<0.001
Rice Krispies^TM^ (18 oz)	51	$7.53	$3.00, 11.38	$5.14	$3.39, 8.54	46%	<0.001
White rice (16 oz)	75	$2.02	$0.50, 3.99	$1.35	$0.50, 2.99	50%	<0.001
White bread (20 oz)	82	$2.20	$0.99, 5.00	$2.74	$0.85, 4.99	−20%	<0.001

Prices were standardized to the size indicated if only another unit or package size was available.

## Supplementary Materials

The following are available online at www.mdpi.com/1660-4601/14/8/915/s1.
