# Supplementary material for: Pricing of Staple Foods at Supermarkets versus Small Food Stores
Source: Int J Environ Res Public Health. 2017 Aug 15;14(8):915. doi: 10.3390/ijerph14080915 (PMC5580618; doi:10.3390/ijerph14080915)
Supplement: Supplementary file 1 [file ijerph-14-00915-s001.pdf]

Supplementary

Minneapolis Staple Foods Ordinance Exclusion Criteria

Per the Minneapolis Health Department website (http://www.minneapolismn.gov/health/living/eating/ staple-foods) licensed grocery stores must meet the staple food requirements. This includes supermarkets, co-ops, and corner stores, as well as many gas stations, dollar stores, and pharmacies. The following types of stores may be exempt from the staple food requirements:

- Accessory use grocery stores (an accessory use grocery store is defined as a retail establishment that sells staple foods as an accessory use to its primary business, or sells only specialized types or classes of staple foods and accessory foods, including, but not limited to, establishments such as imported food stores and gift shops. The definition of accessory use grocery does not include stores that accept government supplemental nutrition programs);
- Gas stations with less than 300 square feet of retail sales area;
- Stores located in the central commercial district (downtown Minneapolis).

Small Food Store Sample

The small food store sample for the study was derived from a list of all licensed grocery stores, acquired from the Minneapolis Health Department for Minneapolis stores and the Minnesota Department of Agriculture for St. Paul stores, with the following exclusions:

**Licensed Grocery Stores in Minneapolis and St. Paul**	**N**
WIC and supermarkets (larger stores)	235
Non-WIC and non-supermarket (smaller stores)	549
Exclusion reason	
Accessory use store	265
Downtown	28
Gas station less than 300 square feet	0
Other (invalid store address, store closed)	31
Stores from which sample was drawn	255
